# Validation of the Dental Age app for automated dental age estimation using Demirjian's method

**DOI:** 10.3389/fdmed.2026.1743600

**Published:** 2026-02-09

**Authors:** Allan Abuabara, Mariana Espindola de Oliveira, Livia Azeredo Alves Antunes, Ellen Cardoso Teixeira, Caio Luiz Bitencourt Reis, Camila Guimarães da Costa Campos, Cristiano Miranda de Araujo, Christian Kirschneck, Flares Baratto-Filho, Erika Calvano Küchler

**Affiliations:** 1 Postgraduate Program in Health and Environment, University from the Joinville Region – Univille, Joinville, Brazil; 2 Postgraduate Program in Dentistry, Health Institute of Nova Friburgo, Fluminense Federal University, Rio de Janeiro, Brazil; 3 Postgraduate Program in Dentistry, School of Dentistry, Fluminense Federal University, Niterói, Brazil; 4 Postgraduate Program in Dentistry, Nova Friburgo Health of Institute, Fluminense Federal University, Nova Friburgo, Brazil; 5 Department of Orthodontics, School of Dentistry of Ribeirão Preto, University of São Paulo, Ribeirão Preto, Brazil; 6 School of Dentistry, Tuiuti University of Paraná, Curitiba, Brazil; 7 School of Dentistry, Tuiuti University of Paraná, Curitiba, Brazil; 8 Department of Orthodontics, University Hospital Bonn, University of Bonn, Bonn, Germany

**Keywords:** age determination by teeth, dental informatics, diagnosis, diagnostic techniques and procedures, mobile applications

## Abstract

**Introduction:**

Dental age estimation is an essential tool in clinical, orthodontic, and forensic contexts, with Demirjian's method (1973) being one of the most widely used worldwide. Recently, digital resources have been developed to automate this process, such as the Dental Age application (Crescendo Treinamentos Avançados, Brazil), available in English for iOS and Android. The aim of this study was to validate the Dental Age app for dental age estimation using Demirjian's method, by assessing its accuracy in relation to chronological age and its agreement with the manual approach.

**Methods:**

This retrospective cross-sectional study used a convenience sample of 63 panoramic radiographs of healthy children treated at a university pediatric dentistry clinic in Nova Friburgo, RJ, Brazil, aged 3–16 years. The mineralization stages of the seven left mandibular teeth were classified by an experienced orthodontist. Dental age was estimated in two ways: (I) manual Demirjian's method and (II) automated analysis using the Dental Age app. Chronological age was used as the reference. Performance metrics (Mean Absolute Error—MAE, Mean Squared Error—MSE, Root Mean Squared Error—RMSE, and coefficient of determination—*R*^2^) were calculated, and Bland-Altman analysis was performed.

**Results:**

The sample included 25 boys (40%) with a mean chronological age of 12.4 years (range: 8.2–15.9) and 38 girls (60%) with a mean chronological age of 12.8 years (range: 7.5–15.9). The Dental Age app showed a MAE of 0.92 years, RMSE of 1.29, and *R*^2^ of 0.63, while the manual method obtained a MAE of 0.91 years, RMSE of 1.30, and *R*^2^ of 0.63. Bland-Altman analysis revealed a mean bias of 0.04 years, indicating a high level of agreement between methods. Intraclass Correlation Coefficient was 0.99, indicating excellent agreement between the Dental Age application and the traditional Demirjian method.

**Conclusion:**

The Dental Age app demonstrated equivalent performance to the traditional method, presenting itself as a practical and reliable tool for dental age estimation in children and adolescents.

## Introduction

The use of mobile applications (apps) and *smartphones* has expanded significantly, representing a technological revolution that directly impacts contemporary society by simplifying and accelerating daily activities (1–3). In healthcare, these technologies have increasingly been applied to support data processing and standardization of clinical procedures, particularly when established methods require multiple manual steps. The health field, particularly Dentistry, has followed this trend through the concept of mobile health (mHealth), primarily by facilitating the automation of diagnostic workflows, which has promoted advancements in diagnostics and radiographic interpretation (2–5).

A patient's biological age, in contrast to chronological age, refers to the degree of maturation of different tissue systems. It can be described from different perspectives, such as skeletal age, morphological age, secondary sexual characteristic age, and dental age. These parameters, used individually or in combination, allow the assessment of the physiological maturity of children during their growth phase (6).

Several methods have been proposed for the assessment of dental age, with the dental maturity scoring system developed by Demirjian et al. being the most widely used (7, 8). In this method, the estimation is based on panoramic radiographs, with analysis of the seven left mandibular teeth, from the central incisor to the second molar. Each tooth is classified into developmental stages, from A to H, with the addition of stage 0 in cases where mineralization is absent. Each stage is assigned a sex-specific numerical value, and the sum of these values yields a score that is subsequently converted into dental age using sex-specific reference tables (7). Despite its extensive use, the Demirjian method has well-documented limitations, including population-specific variability, and systematic overestimation or underestimation of age across different ethnic and geographic groups (8). For this reason, validation studies are recommended whenever the method is applied to new populations or implemented using alternative formats. Furthmore, despite its broad applicability, the manual use of this method requires detailed tooth-by-tooth classification, repeated consultation of scoring tables, and arithmetic conversion steps. This process can be time-consuming and susceptible to calculation or transcription errors, particularly in large clinical samples, or forensic cases requiring rapid and standardized assessments.

In this context, the routine clinical and research demand for timely and standardized assessment of biological maturation highlights the need for automated tools capable of applying established dental age methods with greater operational efficiency. In response to these practical limitations of the manual Demirjian method, the Dental Age mobile application (Crescendo Treinamentos Avançados, Brazil) was developed to automate the scoring and conversion steps. This approach emphasizes improved workflow efficiency, reduced manual errors, and potential support for standardized educational application. Although the app is available on the Apple App Store and Google Play Store and is based on the original Demirjian protocol, the implementation of a traditional method in a digital and automated format may influence measurement outcomes and agreement with the reference standard. Furthermore, despite its commercial availability, no peer-reviewed publications have yet assessed the app's validity, accuracy, or agreement with the manual method.

Therefore, this study aimed to validate the Dental Age app for automated dental age estimation using Demirjian's method, by assessing both its agreement with the traditional manual approach and its accuracy in relation to chronological age, thereby determining whether the automated application provides results equivalent to the reference method.

## Materials and methods

### Ethical aspects

This study was approved by the Ethics Committee of the Universidade Federal Fluminense, under Certificate of Presentation for Ethical Consideration (CAAE) 6.983.655, and was conducted in accordance with the guidelines established by the National Health Council under Resolutions No. 466/12 and No. 510/2016 (9, 10).

### Study design and sample

A retrospective cross-sectional study with a convenience sample was conducted, evaluating panoramic radiographs of healthy children aged 3–16 years treated at the Pediatric Dentistry and Children's Clinic of the Dentistry Program at the Institute of Health of Nova Friburgo, Universidade Federal Fluminense (Nova Friburgo, RJ, Brazil). The developmental stage of the seven left mandibular teeth (excluding third molars) was identified. In cases of dental agenesis or tooth loss on the left side, the corresponding contralateral tooth on the right side was used. Cases with bilateral agenesis or tooth loss, or patients younger than 3 years or older than 16 years, were excluded. Additionally, panoramic radiographs of low image quality were excluded, as such limitations could interfere with proper analysis and compromise the accuracy of the results obtained through the evaluation system.

### Data collection

**Figure 1:**
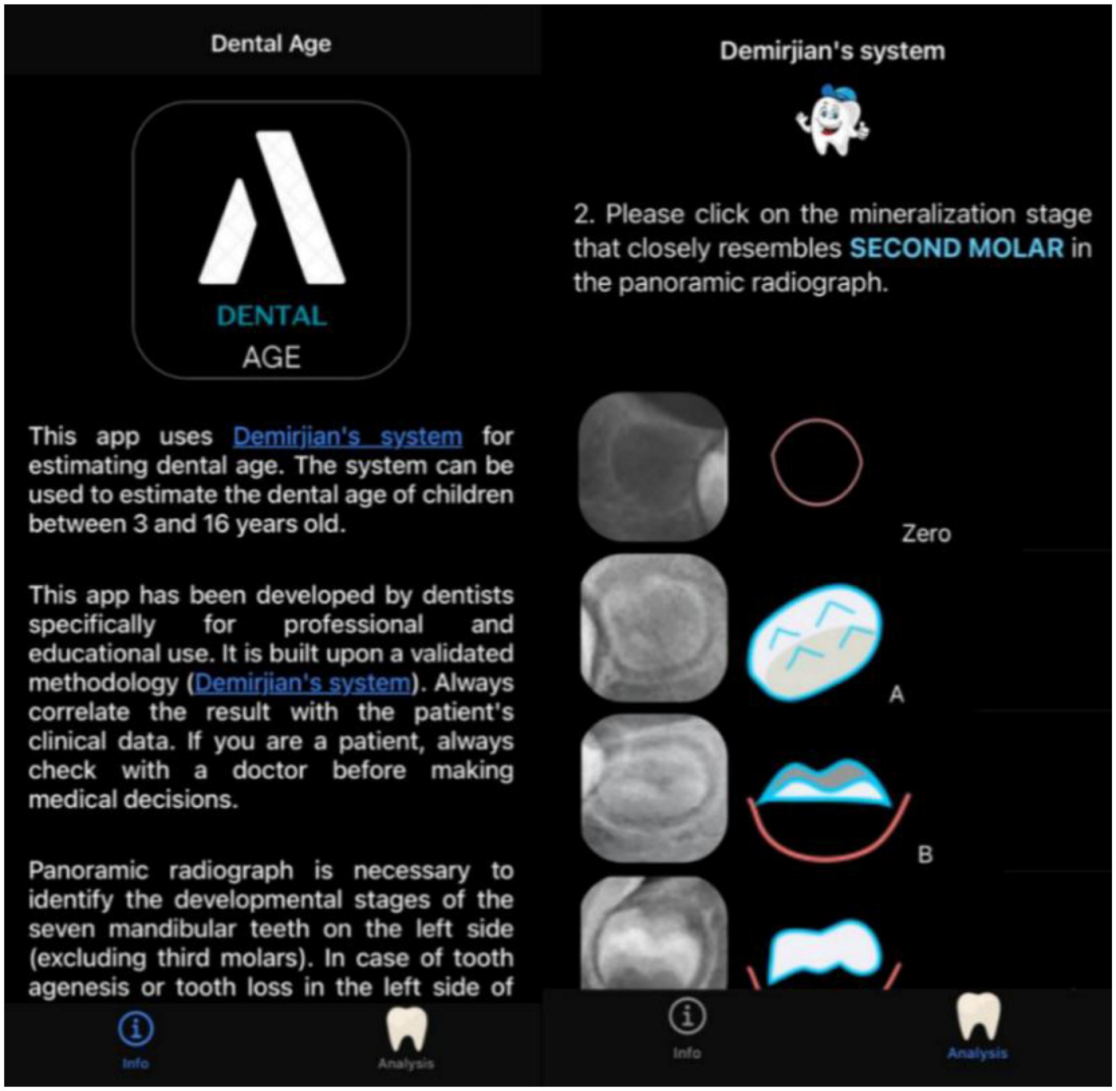
Dental Age application, developed to automate the scoring and conversion steps of the original Demirjian protocol for dental age estimation.

The estimation of dental age was performed using two different approaches: (I) the traditional Demirjian method (7), and (II) an automated analysis using the Dental Age application (Figure 1). Both procedures were carried out by experienced orthodontists (CLBR and ETC). Tooth developmental staging according to the Demirjian scoring system was performed by a single examiner. The same assigned stages (A–H) were subsequently used for dental age estimation by two approaches: manual calculation, performed by the same examiner, and automated calculation using the Dental Age application, in which a second orthodontist was responsible solely for entering the previously defined stages into the app.

Because both methods relied on identical morphological staging, this study specifically evaluated the agreement between manual and automated scoring and conversion steps, rather than examiner-related variability. Consequently, inter- and intra-examiner reliability of tooth staging were not assessed, as observer reproducibility was not part of the research question.

- **Traditional Demirjian method:** This approach consisted of the following steps:
- Identification of the seven permanent teeth of the left lower quadrant (incisors, canine, first and second premolars, and first and second molars) to be evaluated;
- Assessment of the stage of dental development, assigning letters from A to H, ranging from the initiation of calcification (A) to complete development (H);
- Each stage (A–H) received a specific score, differing for boys and girls, based on reference tables published by Demirjian;
- The scores assigned to the seven evaluated teeth were summed to obtain the total dental maturity score;
- The total score was converted into dental age (in years) using sex-specific reference tables or curves (male or female).
- **Automated analysis (Dental Age application):** The Dental Age application applies the Demirjian method through a step-by-step interactive process. First, the examiner selects the patient's sex. Then, for each of the seven left mandibular permanent teeth, the app displays schematic images of mineralization stages (0 or A–H), and the examiner visually inspects the panoramic radiograph to select the stage that best corresponds to each tooth (Figure 1). After all seven stages are entered, the app automatically performs the remaining steps of the original Demirjian protocol—assigning sex-specific scores, summing them to obtain the dental maturity score, and converting this value into dental age.

### Data analysis

Chronological age was used as the reference standard. For each method, errors in relation to chronological age were calculated. Performance metrics included Mean Absolute Error (MAE), Mean Squared Error (MSE), Root Mean Squared Error (RMSE), and the coefficient of determination (*R*^2^), all presented with 95% confidence intervals (95% CI). In addition, agreement between the Dental Age application and the traditional Demirjian method was assessed using the Intraclass Correlation Coefficient (ICC). A two-way mixed-effects model with absolute agreement and single measurements was applied, as it evaluates absolute concordance rather than mere linear association. A Bland–Altman plot was also constructed to evaluate systematic bias and the limits of agreement between methods. An exploratory age-stratified analysis was additionally performed to examine whether the agreement between methods was maintained across different age ranges. The analyses were also stratified by sex. For this purpose, the sample was divided into three age groups (7.5–<11, 11–<14, and 14–15.9 years), and error-based metrics (MAE, MSE, and RMSE) were calculated descriptively for each stratum. This analysis was not intended for formal subgroup comparisons and was interpreted as exploratory. All statistical analyses were performed using the Python programming language in the Google Colab environment, employing the libraries scikit-learn, numpy, and pandas.

### Sample size

The sample size estimation was based on a pilot study of 20 panoramic radiographs, in which a correlation of *r* = 0.76 was observed between chronological age and dental age estimated using the Demirjian method. Using this correlation as a parameter, with a significance level of 1% (*α* = 0.01), power of 99% (*β* = 0.01), and the standard formula for correlation studies, a minimum of 27 participants was required. However, a sample approximately two times larger was intentionally adopted in order to increase the precision of the estimates and enhance the statistical power for agreement analyses (ICC and Bland–Altman).

## Results

Out of a total of 90 panoramic radiographs initially evaluated to ensure the required sample size, 63 met the eligibility criteria and were included in the final analysis. The sample included 25 boys (40%) with a mean chronological age of 12.4 years (range: 8.2–15.9) and 38 girls (60%) with a mean chronological age of 12.8 years (range: 7.5–15.9). The performance metrics showed highly similar results between the two methods. The Dental Age app presented a MAE of 0.92 years (95% CI: 0.70–1.15), MSE of 1.66 (95% CI: 0.89–2.61), RMSE of 1.29 (95% CI: 0.94–1.61), and *R*^2^ of 0.63 (95% CI: 0.38–0.81). Although the error-based metrics were low and comparable between methods, the *R*^2^ value indicates only moderate accuracy, reflecting a limited proportion of variance in chronological age explained by both approaches. The traditional assessment (manual) demonstrated a MAE of 0.91 years (95% CI: 0.69–1.14), MSE of 1.68 (95% CI: 0.90–2.63), RMSE of 1.30 (95% CI: 0.95–1.62), and *R*^2^ of 0.63 (95% CI: 0.36–0.80) (Table 1).

**Table 1:** Performance metrics of the application and the traditional method (manual).

Method	MAE [95% CI]	MSE [95% CI]	RMSE [95% CI]	*R*^2^ [95% CI]
Dental Age app	0.92 [0.70; 1.15]	1.66 [0.89; 2.61]	1.29 [0.94; 1.61]	0.63 [0.38; 0.81]
Manual (traditional)	0.91 [0.69; 1.14]	1.68 [0.90; 2.63]	1.30 [0.95; 1.62]	0.63 [0.36; 0.80]

MAE, mean absolute error; MSE, mean squared error; RMSE, root mean squared error; *R*^2^, coefficient of determination; CI, confidence interval.

**Figure 2:**
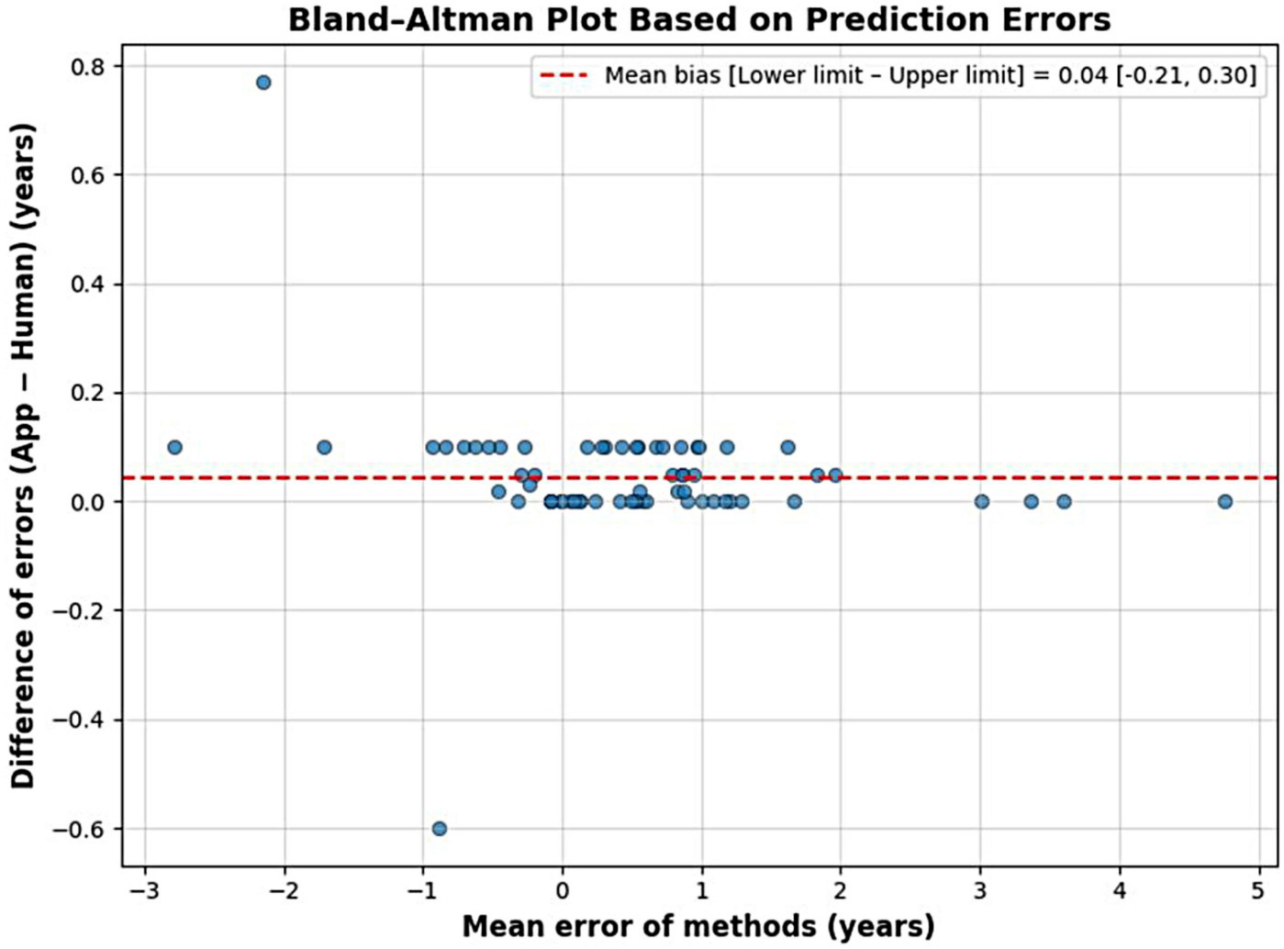
Bland–Altman plot shows the agreement between the Dental Age app and the traditional method, with the mean bias (solid line) and the 95% limits of agreement (dashed lines).

The Bland–Altman analysis revealed a mean bias of 0.04 [−0.21 to 0.30] years, indicating negligible systematic differences between the methods (Figure 2). Most data points were clustered around the mean bias line and within the limits of agreement (±1.96 SD), demonstrating high consistency between the app-based and manual assessments. Only a few isolated values fell outside these limits, with no evidence of systematic overestimation or underestimation. Consistently, the Intraclass Correlation Coefficient was 0.99 [0.98–1.00], indicating excellent agreement between the Dental Age application and the traditional Demirjian method.

To evaluate whether the agreement between methods was maintained across different subgroups, exploratory stratified analyses by age and sex were performed (Tables 2, 3). In the age-stratified analysis, higher error values were observed in the youngest age group (7.5–<11 years) for both approaches, with MAE of 1.26 years for the Dental Age app and 1.24 years for the traditional manual method. In the intermediate age group (11–<14 years), lower and identical MAE values were found for both methods (0.95 years). In the oldest age group (14–15.9 years), the lowest error values were observed, with MAE of 0.62 years for the app and 0.61 years for the manual method.

**Table 2:** Exploratory age-stratified analysis of prediction error.

Age group (years)	*n*	Method	MAE [95% CI]	MSE [95% CI]	RMSE [95% CI]
7.5–<11	13	App Dental Age	1.26 [0.78–1.84]	2.61 [0.93–4.83]	1.61 [0.97–2.20]
		Manual (Traditional)	1.24 [0.76–1.83]	2.56 [0.91–4.80]	1.60 [0.95–2.19]
11–<14	30	App Dental Age	0.95 [0.68–1.30]	1.76 [0.60–3.44]	1.33 [0.78–1.86]
		Manual (Traditional)	0.95 [0.66–1.30]	1.82 [0.59–3.49]	1.35 [0.77–1.87]
14–15.9	20	App Dental Age	0.62 [0.35–0.96]	0.83 [0.25–1.71]	0.91 [0.50–1.31]
		Manual (Traditional)	0.61 [0.34–0.96]	0.84 [0.23–1.79]	0.92 [0.48–1.34]

**Table 3:** Exploratory sex-stratified performance metrics of dental age estimation.

Sex	*n*	Method	MAE (95% CI)	MSE (95% CI)	RMSE (95% CI)
Female	38	App Dental Age	0.92 [0.63, 1.26]	1.88 [0.74, 3.35]	1.37 [0.86, 1.83]
Female	38	Manual (Traditional)	0.93 [0.62, 1.27]	1.96 [0.76, 3.46]	1.40 [0.87, 1.86]
Male	25	App Dental Age	0.90 [0.65, 1.21]	1.33 [0.65, 2.35]	1.15 [0.81, 1.53]
Male	25	Manual (Traditional)	0.87 [0.63, 1.18]	1.26 [0.59, 2.28]	1.12 [0.77, 1.51]

In the sex-stratified analysis, similar error values were observed between the automated and manual approaches within each sex. Among females, MAE values were 0.92 years for the Dental Age app and 0.93 years for the traditional method, while among males, MAE values were 0.90 years for the app and 0.87 years for the manual assessment. Across all age and sex strata, MSE and RMSE values followed comparable patterns between the automated and manual approaches, indicating consistent performance between methods within each subgroup.

## Discussion

This study investigated the agreement of the Dental Age app in estimating dental age compared to the application of the traditional method. Determination of dental age is known to be a relevant procedure in clinical practice, as well as for research and case planning in orthodontics and malocclusion studies. It also has significant applications in forensic dentistry and archaeology (11, 12). In the present study, the Dental Age app produced results comparable to those obtained using the traditional dental age assessment method proposed by Demirjian et al. (7), with performance metrics showing highly similar values between the two methods (app and manual). Moreover, the Bland-Altman analysis revealed a mean bias indicative of no relevant systematic differences. Most validation data points were concentrated within the limits of agreement, demonstrating a high level of concordance between the app-based and human evaluations using the traditional method. These findings indicate that the app indeed provides consistent and reliable results for dental age estimation.

Chronological age does not account for individual variability in biological maturation, which may be influenced by genetic, environmental, nutritional, and socioeconomic factors (13, 14). Consequently, reliance on chronological age alone should be interpreted with caution, as this limitation may directly affect clinical treatment planning in healthcare and compromise age-related assessments in forensic investigations, where accurate estimation of biological maturity is critical. Various methods have been proposed for determining dental age, with the most widely used currently being the method developed by Demirjian et al. (7, 15). This method is well known for its data consistency, ease of standardization, and high reliability (16). Nevertheless, because its reference standards were derived primarily from populations of European origin, its performance may be directly influenced by population-specific growth patterns, leading to systematic bias and reduced accuracy when applied to other populations. The advancement of artificial intelligence and information and communication technologies (ICTs) has led to the widespread use of digital tools, paving the way to enhance existing algorithms in healthcare, such as mobile applications, by improving workflow efficiency and enabling faster and more standardized clinical case planning (17, 18). The use of the Dental Age app to apply this method simplified the process, enabling a rapid and efficient analysis of dental development without the need for complex equipment or software, while preserving the inherent methodological characteristics and limitations of the Demirjian approach.

In this study, the agreement between the app and the traditional method was evaluated. The aim was to verify the accuracy of the results provided by the app, specifically, the degree of proximity to the reference method, to determine its reliability and validity of performance, and to analyze whether its precision corresponds to the level of agreement between the app's estimates and the gold standard (19). Other types of testing, such as usability, performance, and utility assessments, were not the focus of this study (19). The Dental Age app, released in July 2023, can be considered one of the first to initiate the automation process in dental age estimation. There are also studies exploring other methods, such as deep learning–based approaches, which could automate all stages of the assessment (20, 21).

The implementation of digital methods for dental age estimation in clinical settings has increased due to their practicality (18, 20–22). Digital methods have been shown to improve accuracy and reduce inter-examiner variability. These findings underscore the importance of validating automated tools across diverse populations. In the present study, the app maintained the same estimation pattern as the manual method, confirming that automation does not introduce additional bias.

Despite the promising results observed for the Dental Age app, several limitations should be acknowledged. First, although the application automates the scoring and conversion steps of the Demirjian method, the initial classification of tooth mineralization stages remains dependent on examiner judgment. As the present study focused on the agreement between automated and manual implementations of the same scoring system, variability related to morphological staging was not the primary target of analysis and may still influence dental age estimation in both approaches.

Second, this study was conducted using a single-center convenience sample composed of children treated at a university clinic located in a specific region of Brazil. This sampling strategy introduces a potential selection bias, which may limit external validity and restrict the generalizability of the findings (23, 24). Although exploratory stratified analyses by age and sex were performed to assess consistency of performance across subgroups, the limited sample size within strata precludes definitive subgroup inferences.

In addition, the sample size calculation was based on correlation parameters derived from a pilot study and therefore represents an indirect approach for agreement studies. Nevertheless, the use of intraclass correlation coefficients with 95% confidence intervals provides an empirical assessment of precision and helps to mitigate this limitation by quantifying the uncertainty surrounding the agreement estimates.

Regarding external validity, the findings indicate that the Dental Age app demonstrates performance comparable to the traditional manual Demirjian method in terms of accuracy and agreement under controlled conditions. However, clinical, educational, and forensic utility were not directly assessed, and conclusions in these domains cannot be drawn from the present data. Therefore, the results should be interpreted as evidence of methodological equivalence rather than real-world effectiveness. Multicenter studies involving larger and more diverse populations are needed to confirm broader applicability.

Finally, it is important to acknowledge that five of the authors are developers of the Dental Age app. To mitigate this potential conflict of interest, independent authors were included in the study to strengthen methodological rigor and objectivity. Further investigations conducted by fully independent research groups are encouraged to confirm the present findings.

## Conclusion

The Dental Age app showed results comparable to those obtained with the traditional Demirjian method, indicating that it can reproduce the outcomes of the reference approach under the conditions evaluated. By automating the scoring and conversion steps, the application provides an alternative format for applying the method without altering its reproducibility, within the limitations of the study design.

## Data Availability

The data that support the findings of this study are available from the corresponding author upon reasonable request.
